# The Development and Clinical Application of Innovative Optical Ophthalmic Imaging Techniques

**DOI:** 10.3389/fmed.2022.891369

**Published:** 2022-06-30

**Authors:** Palaiologos Alexopoulos, Chisom Madu, Gadi Wollstein, Joel S. Schuman

**Affiliations:** ^1^Department of Ophthalmology, NYU Langone Health, NYU Grossman School of Medicine, New York, NY, United States; ^2^Department of Biomedical Engineering, NYU Tandon School of Engineering, Brooklyn, NY, United States; ^3^Center for Neural Science, College of Arts & Science, New York University, New York, NY, United States; ^4^Department of Electrical and Computer Engineering, NYU Tandon School of Engineering, Brooklyn, NY, United States; ^5^Department of Neuroscience & Physiology, NYU Langone Health, NYU Grossman School of Medicine, New York, NY, United States

**Keywords:** optical coherence tomography, optical coherence tomography (angiography) (OCTA), adaptive optics, visible light OCT, full field OCT, artificial intelligence – AI

## Abstract

The field of ophthalmic imaging has grown substantially over the last years. Massive improvements in image processing and computer hardware have allowed the emergence of multiple imaging techniques of the eye that can transform patient care. The purpose of this review is to describe the most recent advances in eye imaging and explain how new technologies and imaging methods can be utilized in a clinical setting. The introduction of optical coherence tomography (OCT) was a revolution in eye imaging and has since become the standard of care for a plethora of conditions. Its most recent iterations, OCT angiography, and visible light OCT, as well as imaging modalities, such as fluorescent lifetime imaging ophthalmoscopy, would allow a more thorough evaluation of patients and provide additional information on disease processes. Toward that goal, the application of adaptive optics (AO) and full-field scanning to a variety of eye imaging techniques has further allowed the histologic study of single cells in the retina and anterior segment. Toward the goal of remote eye care and more accessible eye imaging, methods such as handheld OCT devices and imaging through smartphones, have emerged. Finally, incorporating artificial intelligence (AI) in eye images has the potential to become a new milestone for eye imaging while also contributing in social aspects of eye care.

## Introduction

No more than four decades ago, images of the eyes were limited to just slit-lamp photographs, fundus photographs, fluorescein angiography (FA), and ultrasounds. Since then, a tremendous growth in technology has given birth to a plethora of techniques to image the eye and has drastically transformed patient care. The invention of optical coherence tomography (OCT) in 1991 was a breakthrough in the field of ophthalmology and has shifted the way patients are managed. Several iterations of OCT are currently utilized, and more are being investigated that would allow researchers and clinicians to obtain a much more in-depth insight into ocular diseases. More recently, evolutions in the field of artificial intelligence (AI) and machine learning (ML), termed by many the “fourth industrial revolution,” have been proved to outperform human evaluations in several aspects, thus providing potential as powerful supplementary tools to help physicians in all aspects of ophthalmic care. The goal of this review is to summarize major advances in the most important aspects of eye imaging, describe novel emerging imaging techniques, and evaluate their use in clinical settings. Hence, imaging modalities widely used in clinical practice (OCT, fundus imaging) and promising imaging techniques (AO) and analysis tools (AI) were selected to be described in more detail.

## Optical Coherence Tomography

Optical coherence tomography was introduced in 1991 (1). It is a non-contact and non-invasive technology that uses low-coherence interferometry, in which a beam from a low coherence interferometer is scanned by moving a reference arm that serves as a reference for depth in the axial direction. In the eye, backscattered light from retinal layers is detected sequentially pixel by pixel to form a depth profile (A-scan) (2). Scanning this beam in the transverse plane creates a B-scan, combining all the acquired A-scans. This provides high resolution two-dimensional images of tissue depth structure and can be applied to the entire anatomy of the eye (retina, optic nerve, cornea, and angle). By collecting a sequential series of B-scans, a three-dimensional image can be produced, with volumetric information on the tissue interrogated.

The first iteration of OCT was time-domain OCT (TD-OCT), which interprets the location of the backscattered light as described above. The image acquisition speed of TD-OCT is constrained by the mechanics of the device; in the first TD-OCT systems, a cross sectional image was acquired in roughly 190 s (3). To address this limitation, high-sensitivity interferometric receivers, optical fiber, and galvanometric beam steering devices were implemented that resulted in an increase in scanning speed (up to 100 A-scans/s), producing the first commercial OCT device, launched in 1996 (4). Further developments in TD-OCT allowed for a maximum speed of 400 A-scans/s (third-generation TD-OCT) (3).

Ten years after the introduction of TD-OCT (2001), new methods of signal acquisition were presented. Fourier-domain OCT (FD-OCT) was the next generation of OCT imaging, with the major changes being that the reference mirror remained fixed, and the frequency spectrum of the reflected light was measured simultaneously and transformed from the frequency to the time domain using Fourier transform. These systems are divided into spectral-domain OCT (SD-OCT) and swept-source OCT (SS-OCT): for SD-OCT, the signals are separated by a grating into different wavelengths, whereas, for SS-OCT (introduced in 2012), the light emitted from the laser source sweeps through frequencies in sequence (3). The center wavelength of these two techniques also differs: most commercial SD-OCT devices use a center wavelength of 850-nm versus 1,050 nm of SS-OCT; the longer wavelengths enable for better penetration and thus improved imaging of deeper structures (5). These implementations allowed for a tremendous increase in scan acquisition speed of commercial devices, from 400 A-scans/s to roughly 100,000 A-scans/s for SD-OCT and more than 200,000 A-scans/s for SS-OCT (5–7). Apart from the scanning speed, these techniques also resulted in improved sensitivity and a signal-to-noise ratio, overall better scan quality, and the ability to perform 3-D imaging.

Optical coherence tomography has become standard in ophthalmology, with multiple applications in diagnosis, monitoring, and management of eye conditions across the board (8). Major advances and implementations in OCT are discussed in the following section, which provide novel and exciting applications in the field of ophthalmic imaging.

### Optical Coherence Tomography Angiography

**FIGURE 1:**
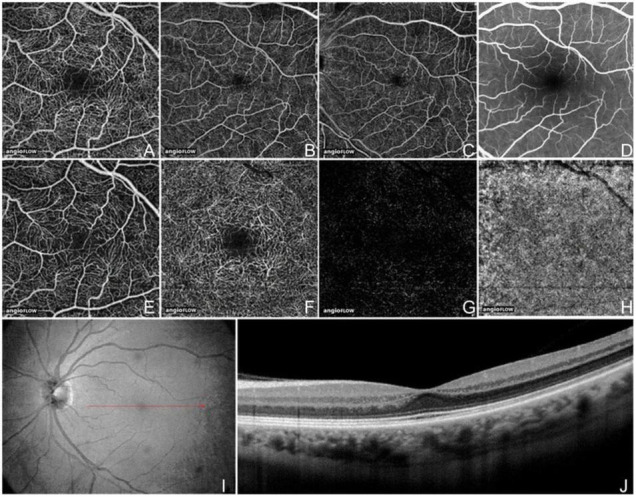
Optical coherence tomography angiography (OCT-A) fields of view and segmentation layers (Angiovue). The normal left eye of a 56-year-old Caucasian man using the Angiovue optical coherence tomography angiography (OCTA) software of the RTVue XR Avanti (Optovue, Inc., Fremont, CA, United States). **(A)** Full-thickness (internal limiting membrane to Bruch’s membrane) 3 mm × 3 mm OCT angiogram. **(B)** Full-thickness 6 mm × 6 mm OCT angiogram. **(C)** Full-thickness 8 mm × 8 mm OCT angiogram. **(D)** Fluorescein angiography cropped to approximately 8 mm × 8 mm or 30 degrees demonstrates a less capillary detail than **(A–C)**. **(E)** 3 mm × 3 mm OCT angiogram of the “Superficial” inner retina. **(F)** 3 mm × 3 mm OCT angiogram of the “Deep” inner retina. **(G)** 3 mm × 3 mm OCT angiogram of the outer retina shows absence of vasculature. The white represents noise. **(H)** 3 mm × 3 mm OCT angiogram of the choriocapillaris is generally homogenous. There is black shadowing from retinal vessels. **(I)** Enface intensity OCT image. **(J)** Highly sampled OCT b-scan image. This figure was reprinted from de Carlo et al. (507) with permission.

Optical coherence tomography angiography (OCT-A) utilizes the motion of red blood cells within blood vessels to image vessels and vascular flow. Commercially available devices developed in 2015 use different algorithms of signal decorrelation from 2 or more repeated B-scans of the same region to display areas of motion: AngioVue (OptoVue, Fremont, CA, United States) uses split-spectrum amplitude-decorrelation angiography (SSADA), whereas Cirrus AngioPlex and PLEX Elite (Zeiss, Dublin, CA, United States) use an OCT-microangiography complex algorithm that is a full-spectrum, complex number-based algorithm (9). The benefits of OCT-A versus other vascular imaging modalities (fluorescent angiography, indocyanine green angiography) are the lack of extrinsic dye injection, leading to adverse effects, and the ability to image vascular networks in different depths (Figure 1). While dye-based angiography can reveal vessel leakage, OCT-A cannot.

Scans acquired with OCT-A can be processed to improve the signal-to-noise ratio (SNR) and display very small vessels more accurately. Denoising methods, such as Gaussian filter (10), compressive sensing (11–20), and Bayesian estimation (11, 21), can suppress noise while maintaining vasculature information. Filters that focus on the specific structure of vessels are also applied, such as Frangi filter (22, 23), Gabor wavelets, and Fuzzy-C-Means Classification (24). Quality limiting factors with OCT-A imaging are the presence of artifacts (projection, motion), visibility of vessels dependent on the rate of blood flow and scan speed, and low output image contrast (25, 26).

The OCT-A can be utilized for conditions involving vascular damage or choroidal neovascularization (CNV) (25). Apart from allowing the subjective identification of vascular abnormalities, OCT-A can also provide quantitative data (vessel density, blood flow, and foveal avascular zone size) that could be employed as vascular biomarkers (27–32).

Diabetic retinopathy (DR) is a disease affecting, among other tissues, the retinal microvasculature (33, 34). It is the leading cause of blindness in the middle-aged and elderly population (35). Early in the disease process, deeper retinal capillary plexuses are primarily affected (36–40). OCT-A can detect both microaneurysms (the hallmark of early DR) and neovascularization (in proliferative disease) in various depths (25, 38, 41–44). Being non-invasive and high resolution, OCT-A can be used for early diagnosis and possible screening of DR when compared to fluorescein angiography (FA) (3, 45). Ong et al. and Russell et al. have proposed new models for DR staging and progression based on OCT-A findings (46, 47).

In the setting of age-related macular degeneration (AMD), OCT-A can detect impaired blood flow at sites of drusen or pseudodrusen (48, 49) and geographic atrophy (GA) (50–52) with high reproducibility when compared to SD-OCT (53). It can also identify CNV (sensitivity, 81% and specificity, ≥93%) with more detail and better contrast than FA (54–64).

In glaucoma, various studies have demonstrated reduced blood flow and blood vessel density at the level of the optic nerve head (ONH) and peripapillary area (65–72). OCT-A scans from macular and ONH scans in glaucoma have been shown to display good reproducibility, which supports the use of OCT-A longitudinally as well (73). Vessel density parameters have also been associated with visual field progression (74, 75).

For other conditions, Zhu et al. have recently proposed that OCT-A metrics can be used to assess and detect myopia development in adolescents (76).

A limitation of OCT-A compared to other vessel imaging techniques is the limited area visualized. Kawai et al. have proposed the introduction of a front prism that would generate ultra-wide field panoramic images and allow imaging of the peripheral chorioretinal vessels, while applying image averaging to correct for the drop-off in image quality (77, 78). Miao et al. also introduced the use of megahertz-rate OCT-A as a faster imaging technique that also yields better contrast images (79). Post-processing methods (angiogram subtraction, distortion correction) and eye tracking during scan acquisition have also significantly improved the scan quality and reduced artifacts (80–86). Furthermore, new types of software have been developed to automate the analysis of OCT-A images; Viekash et al. have also established software to automatically quantify the foveal avascular zone, a region affected by various ocular diseases (87), while tools have been constructed to automatically process, segment, and quantitatively analyze the blood vessels (88–90).

### Visible Light Optical Coherence Tomography

Vis-OCT, first reported by Povazay and coauthors in 2002 (91), has been developed most intensively in the last decade. It relies on light sources from the visible spectrum (555–800 nm), which provides a better axial resolution than typical OCT devices that use near-infrared (NIR) illumination (1.2–1.4 μm versus 1.7–7.5 μm, respectively) (92–96). Other benefits of Vis-OCT include a smaller bandwidth to achieve the same resolution, leading to easier dispersion compensation, and higher image contrast due to higher scattering coefficients (97). One of the advantages of Vis-OCT is the ability to measure oximetry in blood vessels. Blood, which contains oxyhemoglobin (HbO_2_) or deoxyhemoglobin (Hb), exhibits more contrast at the isosbestic point in the wavelengths of Vis-OCT than in the NIR. Combining this property with simultaneous measurement of the blood flow rate using Doppler OCT methods, many metabolic parameters of retinal circulation can be extracted, such as O_2_ saturation (sO_2)_, O_2_ extraction fraction, total retinal O_2_ delivery, and the metabolic rate; Vis-OCT has been validated and is superior to NIR-OCT for these parameters (97–103).

Vis-OCT has been widely studied in disease models in animals and is now transitioning to clinical practice. Rodents with DR and retinopathy of prematurity (ROP) have been studied using Vis-OCT, and metabolic changes in both conditions were apparent before structural changes (103–105). The first use on humans was performed by Yi et al. on a single healthy subject, with Vis-OCT displaying increased contrast of the inner (the retinal nerve fiber layer – RNFL) and outer (a photoreceptor inner/outer segment, a retinal pigment epithelial layer, Bruch’s membrane) retinal layers compared to NIR-OCT (106). Vis-OCT has since been used for calculating metabolic parameters with upgraded light sources, decreased noise, and great axial resolution (96). Shu et al. also developed a Vis-OCT platform than can be used for humans and demonstrated it successfully in conditions such as retinal occlusive diseases and DR (107). Given its ability to provide metabolic information about oxygenation and circulation, and its advantages of retinal layer imaging over NIR-OCT, Vis-OCT might be a better imaging candidate for conditions affecting these cell layers, for example, the RNFL, inner plexiform layer (IPL), and inner nuclear layer (INL) in glaucoma or the outer retinal layers and retinal pigment epithelium (RPE) in AMD. Its ability to provide metabolic information about oxygenation and circulation, contributors to retinal and optic nerve diseases, reinforces this point.

**FIGURE 2:**
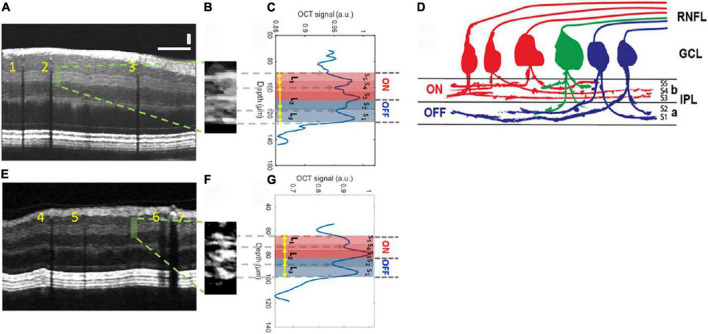
Inner plexiform layer (IPL) sublayer visualization with Vis-OCT. **(A)** A speckle-reduced vis-OCT image from a healthy eye. A horizontal bar: 500 μ m; a vertical bar: 50 μ m. **(B)** A magnified view of the region highlighted by the dashed box in **(A)** (15 srA-lines segments). **(C)** A depth-resolved OCT amplitude profile of the IPL sublayers. We averaged 15 srA-lines, corresponding to approximately 88 μ m along the lateral direction within the highlighted region in **(A)**. **(D)** Illustration of the lamination of ganglion cells from RNFL to the IPL. The “red” ganglion cells (ON center) are laminating dendrites to the “b” sublamella of the IPL whereas “blue” cells (OFF center) laminate to the “a” sublamella. The “green” ganglion cell is bi-laminating. **(E)** A speckle-reduced vis-OCT image from a glaucoma eye. **(F)** A magnified view of the region highlighted by the dashed box in **(E)**. **(G)** A depth-resolved line profile of the glaucoma eye IPL sublayers. This figure was reprinted from Ghassabi et al. (113) with permission under a Creative Commons Attribution 4.0 International License.

Vis-OCT has continued to develop. Registration and averaging of multiple volumes have allowed the visualization of single cells, and the concept of Vis-OCT fibergraphy (Vis-OCTF) of imaging-specific retinal ganglion cell (RGC) axon bundles has been introduced in animal models (108–110). Ultrahigh resolution Vis-OCT (UHR Vis-OCT) is capable of imaging sublayers within retinal layers, such as the IPL (Figure 2), and improvements in the axial resolution have also been made using rapid spectral shaping, axial tracking, and *in vivo* spatially dependent numerical dispersion compensation (111–113). Zhang et al. have also developed a circumlimbal scanning method to image the anterior segment, capable of visualizing the Schlemm canal and the limbal microvascular network (114). In addition, Wang et al. have recently devised a dual-channel system with both Vis-OCT and NIR-OCT-A capable of simultaneous retinal imaging and metabolic measurement acquisition; the use of a fiber-based dual channel Vis- and NIR-OCT to image the human retina was first reported by Song et al. (115, 116). In a clinical setting, this dual channel system was initially reported to quantify RNFL spectroscopic markers in glaucomatous subjects and glaucoma suspects, which were correlated with disease severity (117). A combination of both Vis-OCT and OCT-A (Vis-OCT-A) has also been reported by Song et al., with narrow bandwidth spectrometers, optimized image protocols, and improved acquisition speed (100,000 A-scans/s) (118).

### Handheld Optical Coherence Tomography

In a typical OCT imaging session, the patient sits upright with the head stabilized with a chin rest and headrest. The use of portable or handheld OCT is indicated in cases where this positioning is not possible. Populations included in this category are bedridden or postoperative patients, the pediatric population and cases where access to health care is limited or difficult. Portable HH-OCT would potentially offer an easier-to-use and less-expensive alternative to current commercial OCT devices. Chen et al. displayed that measurements from HH-OCT have good repeatability and reproducibility of both axial and transverse measurements when compared to Heidelberg Spectralis (119).

The HH-OCT is widely used nowadays for pediatric ocular conditions. HH-OCT/OCT-A systems can reliably visualize and measure vitreous opacities and bands, perifoveal vessels, the macular shape, anterior chamber features, retinal tumors, and ganglion cell complex (GCC)/IPL/RNFL thickness in both premature and full-term infants (120–130). Hence, they are useful in conditions, such as congenital and pediatric glaucoma, macular edema, macular hole, epiretinal membrane, retinoschisis, retinal dystrophies, and other conditions (128, 129, 131–133). Some measurements obtained, mostly from the fovea, could potentially be used to screen for conditions like ROP (132). HH-OCT-A in specific is extremely useful in cases of CNV in children, such as ROP, retinal dystrophies, inflammatory disorders, trauma or cases of unexplained visual loss (134–136). Ocular measurements, specifically RNFL thickness, have been shown to be associated with systemic health conditions in infants (low birth weight, sepsis, and necrotizing enterocolitis) (137).

**FIGURE 3:**
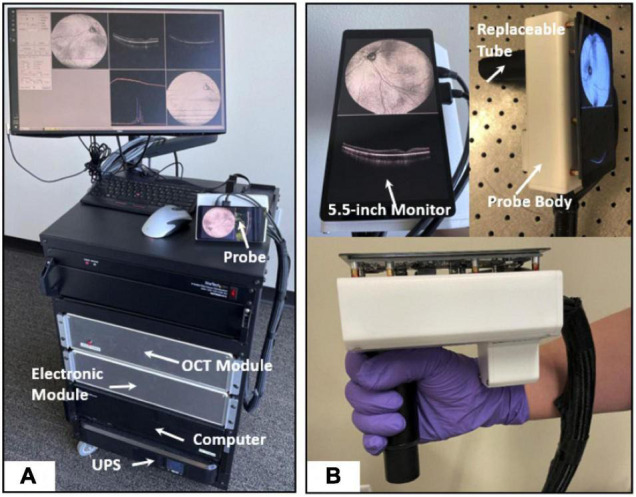
High-speed and widefield handheld SS-OCT-A with a VCSEL light source. **(A)** A photograph of the front of fully assembled handheld OCTA system in a portable cart. **(B)** A photograph of the handheld probe. This figure was reprinted from Ni et al. (151) with permission.

The approaches to making OCT devices more mobile are promising. HH-OCT probes have been developed and are currently used in animal experimental models (Leica, Heidelberg); this allows for transportation of the device, although the probes are tied to bulky mobile carts (138, 139). Smaller versions of OCT machines have also been achieved that offer comparable results to bench-top OCT devices, with some offering *en face* reflectance, OCT-A volumes, combination with SLO and supine imaging (140–148). Handheld SS-OCT devices have also been created, containing ultrahigh speed and averaging capabilities (Lu et al.) or capable of imaging both the anterior and posterior segments in quick succession (Nankivil et al.) (149, 150). Ni et al. have recently proposed models for high-speed scanning (the HH-SS-OCT model using a 400 kHz VCSEL light source, scanning speed, 1,720 MHz; volume acquisition time, 1.875 s) and an increased field of view scanning to 105 degrees (Figure 3) (151, 152).

### Intraoperative Optical Coherence Tomography

Intraoperative OCT (I-OCT) can aid surgeons by providing a live imaging feedback during surgery. The devices currently used for I-OCT are either handheld or integrated in microscopes or probes (153). HH-OCT in this setting is best utilized when mounted onto the surgical microscope to improve stability and precise movement. The images obtained with that method are fast, accurate, and reproducible; drawbacks include pausing the surgery for image acquisition and potential requirement of technician assistance (154). I-OCT integrated within the surgical microscope partially resolves these issues, as it can be used without pausing the operation or additional specialized technical support; additionally, there are opportunities to enhance I-OCT with decision-making algorithms and high-tech instrumentation (e.g., heads-up display) (155). An advanced technique is implementation of OCT scanners in ophthalmic probes, creating an instrument for intraocular use, especially for vitreoretinal surgery (156).

The I-OCT can provide insight into diagnosis and surgical planning, optimal outcome confirmation, complication prevention and control, prognosis, and education. Ehlers et al. in two studies (PIONEER and DISCOVER) reported that I-OCT findings can affect surgical decision-making in 29–68% of select surgery types (154, 157). For epiretinal membrane peeling, I-OCT can precisely locate the margins of the membrane, dictate the best start and end points for peeling, and confirm successful peels without further complications (a point of disagreement between subjective observation and I-OCT) (154, 157–160). In macular hole repairs, I-OCT can confirm the release of traction, effectiveness of the tamponade or flap and hole closure; on the latter, Kumar et al. recognized residual tissue at the hole edge (a “hole-door” sign) as an imaging factor predicting the rate of hole closure (161–166). Recently, Cehajic-Kapetanovic et al. have reported to be the first group using I-OCT to guide a robot-assisted drug delivery during vitreoretinal surgery; in that context, I-OCT can be used to guide all kinds of retinal and subretinal treatments, including highly promising gene therapies for retinal conditions (167–169).

In surgeries of the anterior segment, the ease of imaging of the cornea makes it an attractive I-OCT target (153). For deep lamellar anterior keratoplasty (DALK), where the corneal stroma is dissected, I-OCT allows the surgeon to evaluate the depth of the dissection, make on-the-spot adjustments, and confirm layer separation and integrity of Descemet’s membrane (153, 170–173). Implementations of I-OCT in DALK have been shown to lead to successful outcomes (171, 174, 175). Posterior corneal procedures like Descemet stripping (automated) endothelial keratoplasty (DSEK/DSAEK) and Descemet membrane endothelial keratoplasty (DMEK) can also benefit from the use of I-OCT. The handling, unfolding, and positioning of the graft can be performed more quickly and definitively with simultaneous I-OCT, which can also verify its correct orientation (176–181). Apart from this, fluid between the cornea and the graft (interface fluid) and areas of graft non-adherence or folds can be assessed and addressed (176, 182–184). In the setting of cataract extraction surgery, I-OCT could be beneficial for identifying and handling complications, confirming adequate placement of the lens and further improve the accuracy of refractive calculations and aid in the development of future lens designs (153, 185–187).

### Whole-Eye Optical Coherence Tomography

Different parts of the eye require imaging configurations specific to the area examined (anterior segment versus retina), mainly due to different natural properties. For anterior segment, imaging light has to pass only through the air to the tissue of interest versus the refractive structures (cornea and lens) for retinal imaging. The scan depth using standard OCT is typically about 2 mm, well below the typical axial length of the eye (188). Whole-eye OCT offers the opportunity to acquire a view of the eye from anterior to posterior segments with a single scan.

Multiple approaches have been implemented for that goal. Commercial systems (Heidelberg Spectralis, OptovueiVue, Leica Bioptigen C-series) are able to scan 2 areas in sequence by changing the scan configurations, for example, the reference arm length. In addition, changing the imaging optics (by adding lenses or using an adjustable lens) or alternating the volume frames helps decrease the differences of the structures imaged (150, 189–191). The main drawback of switching scan configurations is the time gap for changing the settings. Recently, Luo et al. have demonstrated an SS-OCT prototype that utilizes a single source and a single detection channel for sequential imaging (192).

Newer approaches of whole-eye OCT aim to capture images of all structures at the same time and be true whole-eye scanners. Approaches include the use of 2 practically separate subsystems (the first for anterior segment and the second for retina) or a single system with either one or two different imaging depths (193–196). The latter is the most advanced method, with the dual-depth polarization system focusing on both structures at the same time, using either one or two interferometers (197, 198). Its big advantage is the focusing of each area while also achieving standard fields of view greater than 24 degrees to image both the macula and the ONH.

In clinical application, whole-eye OCT can provide biometric data for the entire eye (axial length and lens thickness); this, along with information about possible retinal comorbidities and/or microstructural lenticular changes (for example, posterior capsule integrity), can be useful in planning of refractive or cataract surgeries (199–202). Following cataract extraction surgery, it can also verify correct lens positioning and capsule integrity (202, 203). Moreover, visualization of the entire eye could be relevant to patients with high myopia and potentially provide insight into possible causes of pathologic myopia. In certain conditions involving multiple ocular structures, such as the anterior chamber angle and the RNFL in glaucoma, whole-eye OCT could provide data for multiple regions of interest (204).

### Anterior Segment Optical Coherence Tomography

The interest of imaging the anterior segment became apparent soon after OCT’s introduction in 1991. A decade and a half later, OCT systems designed specifically for that purpose were developed that utilized TD-OCT [Visante (Carl Zeiss Meditec, Dublin, CA, United States; 2005), Slit-Lamp OCT (Heidelberg Engineering GmbH, Heidelberg, Germany; 2006)] or SS-OCT [Casia SS OCT (Tomey, Nagoya, Japan); 2008] and were able to image the entire anterior segment.

**FIGURE 4:**
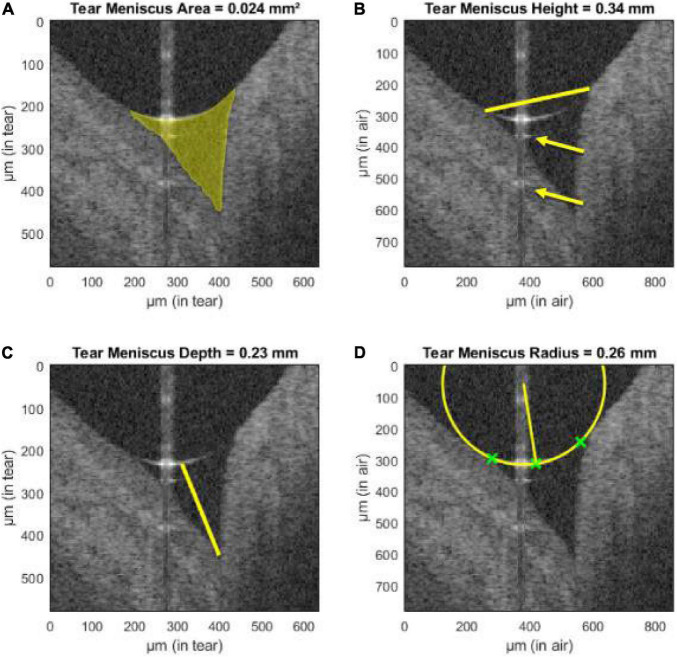
Assessment of tear meniscus using UHR-OCT. Automatic segmentation of the lower tear meniscus in a healthy subject. Calculated parameters (represented in yellow) are **(A)** the tear meniscus area, **(B)** height, **(C)** depth, and **(D)** radius of curvature. Green crosses represent the points used for the estimation of the radius of curvature. The yellow arrows indicate mirror artifacts of the true upper meniscus boundary due to internal reflectors in the optical setup of the system. This figure was reprinted from Stegmann et al. (209) with permission.

The AS-OCT can be a tool in diagnosing conditions involving the anterior segment. A common condition is dry eye disease (DED), in which diagnosis can be a challenge due to poor association of corneal findings and actual symptoms (205). AS-OCT can measure the precorneal tear film thickness and individual tear film layers (lipid and aqueous) and the tear meniscus (area, height, depth, and radius) (Figure 4); these parameters correlate with objective corneal findings, subjective symptoms, and other tests used in DED (such as the Schirmer test) or can be used to evaluate treatment response and monitoring (206–214). For other corneal pathologies, AS-OCT can also be an aid in diagnosing various types of keratitides (fungal, viral, bacterial, and parasitic), ocular surface neoplasias, corneal edema, corneal dystrophies, differentiate pterygium from pseudopterygium and assessing keratoconus morphology (95, 215–223).

A special utilization of AS-OCT is in the setting of glaucoma research. Elevated intraocular pressure (IOP) in primary open angle glaucoma (POAG) is due to increased resistance in the aqueous outflow system, and AS-OCT can be used for evaluation and for better understanding of the pathophysiology. In POAG, the decreased Schlemm canal cross-sectional area has been reported compared to healthy subjects (224, 225). AS-OCT can also visualize the anterior chamber angle qualitatively and quantitatively (angle opening distance, angle recess area, and trabecular-iris space area), with these findings correlating well with ultrasound biomicroscopy (226, 227). Baskaran et al. reported the correlation of angle closure on AS-OCT and gonioscopic angle closure (228). Risk factors for angle closure can be identified using AS-OCT, such as iris thickness/area, anterior chamber width, lens vault, and anterior chamber area/volume (229–231). Further AS-OCT advances would allow better understanding of the inciting events of angle closure glaucoma. The response of the trabecular meshwork to elevated IOP can be visualized and quantified (232). In the laboratory, using automated software, a 3D reconstruction of the entire SC and collector channels is now possible; changes in these structures can help in guiding glaucoma surgeries and predict or monitor IOP-lowering treatment success (both medical and surgical) (233–237). Ruggeri et al. have recently combined AS-OCT with a wavefront-based aberrometer, which was capable of using the OCT beam to acquire refractive error measurements and allow for simultaneous imaging, autorefraction, and biometry (238).

Recent advances in the field of OCT have also been applied to modern AS-OCT, namely, increased scanning speeds (up to 2 million A-scans/s), greater depth, and improved axial resolution (up to 1 μm) (239–246). These have allowed the imaging of most structures of the anterior segment, including all the corneal layers and the precorneal tear film, the outflow system (trabecular meshwork, Schlemm canal, collector channels, and scleral veins), and the anterior chamber angle (247–250).

### Full-Field Optical Coherence Tomography

In FF-OCT, a light emitting diode is used to illuminate the entire scanning field simultaneously, which captures images orthogonal to the optical axis (en face) and avoids transverse scanning (251). Challenges to FF-OCT include eye movements and difficulties matching of the length of the optical path; cameras with high acquisition speeds and combinations with TD-OCT and FD-OCT aim to resolve these issues. Although time-domain FF-OCT (TD-FF-OCT) is possible, its slow volumetric capture capability limits is use (252). Fourier-domain FF-OCT (FD-FF-OCT), on the other hand, can capture 3D volumes of the cornea and retina with scanning speeds reaching 38.6 MHz (253). The single phase of FD-FF-OCT across the entire field does not introduce motion artifacts seen between A-scans in conventional OCT and allows for the use of higher scanning power, leading to fewer aberrations and less signal loss (252, 254).

The FF-OCT can be used to image and study structures of the anterior segment. Mazlin et al. presented the first FD-FF-OCT system capable of corneal imaging the corneal epithelium, stroma, and endothelium (255). The same team later created a system, combining FF-OCT with SD-OCT capable of cellular level imaging of the entire ocular surface (256). Using a common path (NIR light-emitting diode), the SD-OCT arm was used to track axial and lateral eye movements and adjust the optical arm lengths of the FF-OCT accordingly. This allowed for *in vivo* detailed imaging of all the corneal layers, sometimes even to the cellular level (superficial epithelium and stromal keratinocytes), including nerve plexuses. Apart from the cornea, quantitative parameters from structures, such as the tear film (tear flow velocity, amount of particulate matter, and evaporation time after blinking) and the corneal limbus (blood vessel morphology, blood flow velocity, and blood flow direction), were also measured. These can be useful for research studies of relevant anterior segment pathologies (DED, anterior chamber inflammation, and conditions leading to scarring). This approach is comparable to confocal microscopy, as it images the same corneal microscopic features much faster, in a non-contact manner and from a broader (nine times larger) field of view, with high axial resolution and without the requirement of fluorescein administration. These features make FF-OCT an excellent candidate for *in vivo* histological studies without sample preparation.

A similar approach with combination of FF-OCT and SD-OCT has been used for retinal imaging. A device using that principle was presented by Xiao et al., which was able to image the RNFL and photoreceptor layer in great detail (orientation of nerve fibers and cone photoreceptor mosaic, respectively) (257). These images were comparable to AO-OCT commercial devices (discussed below in greater detail), although the SNR was lower in the photoreceptor layer and not adequate to image other retinal layers, such as the ganglion cell layer and RPE; visualization of layers also required averaging of multiple images after acquisition. Other teams have also succeeded in imaging the photoreceptor layer with FF-OCT (258). For remote retinal scanning with FF-OCT, von der Burchard et al. have also proposed a low-cost device, which patients can use to examine themselves and monitor disease progression from their homes (259).

## Adaptive Optics

By using wavefront technology first utilized in astronomy and defense systems, adaptive optics (AO) systems have been implemented in ophthalmology with the goal of correcting for ocular anatomical and physiological higher order (optical/wavefront) aberrations (cornea, lens, and pupil), which cannot be corrected by glasses, contact lenses or refractive surgery (260). As a result, AO vastly improves the transverse (lateral) resolution as well as the speckle width and increases the SNR *via* imaging through a larger pupil, allowing more light to enter the eye. Further improvements in the lateral resolution have also been made by the addition of mirrors and error budget analyses (261–264). AO has so far been implemented in multiple imaging modalities, such as OCT (AO-OCT), scanning laser ophthalmoscopy (AO-SLO), and two-photon ophthalmoscopy (AO-TPO) (265, 266).

**FIGURE 5:**
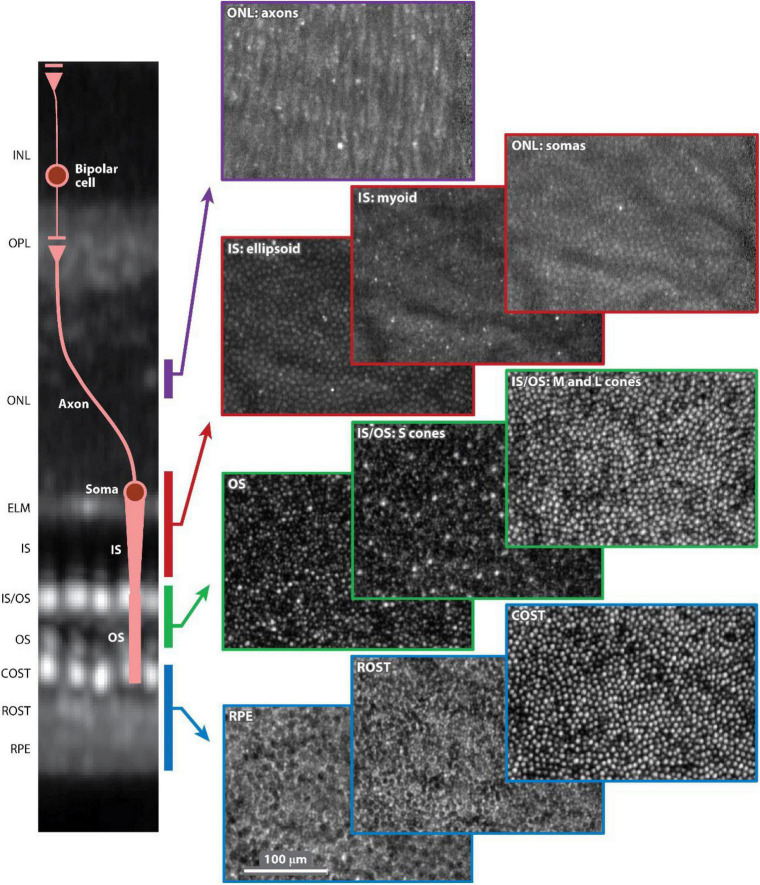
Adaptive optics optical coherence tomography (AO-OCT) volume image of the outer retina of a 52-year-old normal subject. Ten en face (C-scan) images are shown selected from the volume and color-coded by depth in the outer retina, as denoted in the cross-sectional slice (B-scan) on the left. Each C-scan image is normalized to itself and presented on a log intensity scale. The AO-OCT volume image is an average of approximately 2,200 registered volumes that were acquired at 3.7° temporal to the fovea. AO-OCT, adaptive optics optical coherence tomography; COST, cone outer segment tip; ELM, external limiting membrane; INL, inner nuclear layer; IS, inner segment; IS/OS, inner segment/outer segment junction; ONL, outer nuclear layer; OPL, outer plexiform layer; OS, outer segment; ROST, rod outer segment tip; RPE, retinal pigment epithelium. This figure was reprinted from Miller et al. (262) with permission.

The improved lateral resolution of AO systems has enabled the visualization of single cells within the retina (Figure 5). By combining this feature with the high axial resolution of OCT, a resolution voxel smaller than most retinal cells is acquired: the lateral resolution achieved (2–3 μm) is about 5 times higher and overall resolution 36 times greater than commercial OCT (267–272). The big advantage, therefore, of AO is the ability to track cellular changes over time either for studying disease processes or monitoring treatment responses, both of which could be very useful clinical applications.

**FIGURE 6:**
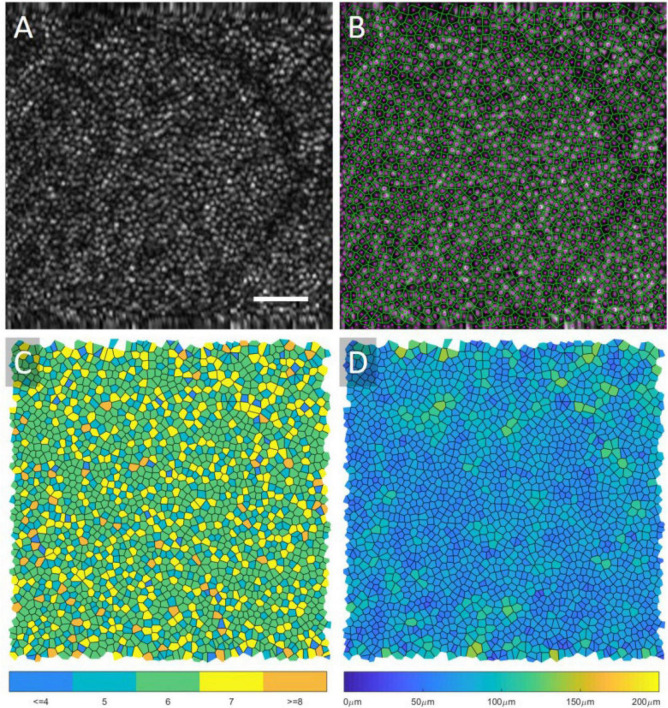
Voronoi analysis of photoreceptors from AO-OCT. An original AO-OCT image taken at ∼6.5° retinal eccentricity is displayed in **(A)**, and the center of the cones (magenta) and the Voronoi map (green) is overlaid onto the image in **(B)**. In **(C)**, the Voronoi cells are shaded based on the number of neighbors, and, in **(D)**, the cells are shaded based on their area. A scale bar, 50 μ m. This figure was reprinted from Heisler et al. (508) with permission.

Cone photoreceptors are the cells most widely studied using both AO-OCT and AO-SLO. Anatomically, they have been shown to possess a wide range of reflectance properties, and their major components (inner segment, outer segment, somas, and axons) can be visualized, with results corresponding to histology. They can also be functionally distinguished by their light sensitivity in different types (short, medium, and long wavelengths). Photoreceptors are normally organized in a lattice hexagonal pattern in the retina, and their architecture can be extracted by semi-automated segmentation methods (273, 274). After identification, mapping the structure formation (Voronoi analysis) allows for descriptive quantitative measurements (cone density, cone spacing, and mosaic regularity), which can be utilized with good inter-device reproducibility (2.5–6.9%) as biomarkers or used to construct normative databases for research or clinical practice (Figure 6) (275–278). The calculation of functional biomarkers (cone reflectance) is also possible (279). Rods are more difficult to visualize than cones since they have a smaller diameter (especially peripherally), different refractive indices, and greater interference with the RPE (280). Despite these drawbacks, AO-SLO studies using improved AO systems have demonstrated visualization of rods in both normal and diseased subjects (281).

Imaging of several retinal cell types is possible with AO. These include individual RNFL bundles and sublayers of the ONL, which has been shown to consist of two distinct sublayers (somas and axons) (262, 269). These are mostly visible with AO-OCT, since the limited axial resolution of AO-SLO cannot easily image the transparent, multi-cell thick inner layers (280). Confocal AO-SLO, in which light is focused on a single spot and the backscattered light is refocused on a confocal aperture, is capable of imaging some of these structures (photoreceptor segments and nerve fibers), as well as blood vessels and the lamina cribrosa (282). AO-SLO-FA can also capture blood flow and detect vascular leaks. Other non-confocal detection modes (offset aperture, dark-field, split-detection, and offset pinhole) and the implementation of multi-volume averaging have also been implemented in order to visualize the ganglion cells and RPE (277, 283–285).

These features allow a detailed study of the retina and its cellular microstructure both in normal eyes and in ocular pathologies, making AO a useful adjunct to other OCT imaging technologies. In AMD, retinal layer and RPE changes can be monitored in areas of drusen/pseudodrusen or GA (286). Panorgias et al. have demonstrated both losses in reflectivity between the photoreceptor inner and outer segments in areas of GA (287). This could be extremely helpful in determining the progression of events in AMD and determining which tissues are affected first during the disease onset (277, 288).

Decreased cone density in areas of RNFL thinning was also displayed in glaucoma and other neuropathies (289, 290). Multiple groups have demonstrated that RNFL bundles are lost in areas of scotomas in glaucoma, and that the polarization properties change in the disease course (270, 271, 291). The increased transverse resolution of AO-OCT can also be utilized to better visualize the microstructure of the lamina cribrosa in glaucoma, a site of early damage, and, therefore, aid in earlier diagnosis (291).

The 3D representation of retinal microvasculature with AO-OCT can also make it a valuable tool in the assessment of DR, especially in combination with OCT-A, for study of microaneurysms, vessel tortuosity, and capillary dropout (270, 292–295). Decreased cone density has also been reported to be 10% lower in DR, which could suggest a method for earlier detection (296).

Finally, the cellular identification of either AO-OCT or AO-SLO can be used to study disease mechanisms of inherited color defects and monitor treatment response in stem cell transplantation therapies for said retinal diseases or central serous chorioretinopathy (280, 297, 298). For treatment of retinitis pigmentosa specifically, multiple AO modalities (AO-OCT and AO-SLO) can be used to stage the disease based on individual cell health, assess visual function from the cellular structure, and select candidates that could benefit from treatment (299, 300).

The application of AO-OCT in routine clinical settings is still challenging, mostly due to high cost, size, and complexity of the devices, time-consuming image acquisition and analysis, a limited scan area (generally no larger than 200 μm × 200 μm transversely), data size, and the lack of established normative databases (280). Some additional limitations are particularly present with specific populations who would benefit from AO applications, for example, unstable fixation in young children with inherited retinal dystrophies. For these reasons, AO-SLO and AO-OCT remain primarily research tools but are applicable to a number of ongoing clinical trials (301).

Despite these challenges, there have been developments over the last few years in the AO systems. Reumueller et al. have recently though developed AO-OCT prototypes that are promising and can be applied in patients with the aforementioned pathologies (302, 303). Improvements over the standard AO-SD-OCT, such as point-scanning sensorless SD-OCT, computational AO, and line-scanning SD-OCT, are currently being investigated (277, 304, 305). The acquisition speed of images has been improved by various groups up to 1 million A-lines/s, the fastest retinal SD-OCT at the time, while the implementation of AO in SS-OCT systems allows for even higher speeds (262, 269, 291, 306–308). The increased scanning speeds have aided in resolving the issue of motion artifacts, an issue also addressed by means of active retinal tracking (integration of AO-SLO with wide field or tracking SLO) and registration of multiple scans (306, 309–314). The registration and image averaging of scans also allows for temporal tracing of cellular processes in time and improves the SNR and contrast, which make individual cells more apparent; examples include better visualization of the RPE, RGC somas, IPL bands, and hyalocytes on the internal limiting membrane (ILM) (262, 285, 315–319). These cellular parameters are attractive, as they can be used as biomarkers for screening or longitudinal follow-up. As an example, AO-SLO can detect cell destruction in retinal degenerations before the onset of symptoms, which would allow the formation of therapeutic clinical trials, while vision is still salvageable in these patients (280, 320). At the same time, improvements in the axial resolution of OCT devices *via* increasing the bandwidth of imaging sources (greater than 100 nm) have allowed the development of AO ultra-high-resolution OCT (AO-UHR-OCT) (267). Pandiyan et al. implement techniques (increased source bandwidth, improved Nyqyist sampling, increased illumination beam size at the pupil, spherical mirror-based telescopes instead of lens-based telescopes, optimized design software and tools) that improve the resolution of both structure and function, achieving visualization of both individual foveal rods/cones in *en face* projections and RGCs; this has also been achieved by other groups (321, 322).

### Phase-Sensitive Optical Coherence Tomography and Optoretinogram

The AO has the potential to capture images of single cells from a snapshot in time. Assessment of the temporal function is also possible through the development of phase-sensitive OCT. It is known that the reflectivity of retinal cells, caused by photoisomerization of pigment chromophores, can vary after application of visual stimuli (optical phase changes) (323). Measurement of these changes is possible with either OCT, hence the term “phase-sensitive OCT” (324). This is the foundational basis of the optoretinogram (ORG), which allows recording of responses to visual stimulation. The ability to detect the function of individual photoreceptors would allow detection of retinal dysfunction in a microscopic scale. As opposed to OCT assessment, where the result is binarized (alive versus lost photoreceptors), ORGs provide a spectrum of function and identify cells that could be salvaged (280).

The implementation of ORG in a clinical setting still has some drawbacks. Studies, so far, have only focused on healthy individuals or patients without severe conditions, whose imaging might pose challenges, such as poor fixation, pathologies hindering image quality, and long periods of dark adaptation not easily tolerable. Furthermore, even after image acquisition, processing of these images is hours long. Despite these difficulties, ORG can potentially be implemented in other devices currently used (SLO and AO-SLO, OCT/OCT-A and AO-OCT) with minimal additions (a stimulus channel and appropriate software) to provide a functional component to structural measurements.

## Artificial Intelligence and Integrated Machine Learning

The implementation of AI in the field of ophthalmology has been a revelation. Ophthalmology offers a great basis for AI to grow and be utilized, owing to the combination of data availability for highly prevalent conditions (glaucoma, AMD, and DR), which are always rising as the population ages, developments in teleophthalmology, and the reliance of these conditions on low-cost, easily performed images (predominantly fundus images and OCT) (325). AI strives to solve some major obstacles in many aspects of eye care: It can provide valuable feedback for diagnosis, monitoring and follow-up, correct treatment decisions, and prediction of disease course. These benefits are especially relevant to conditions requiring highly specialized care by experts; its use by comprehensive ophthalmologists can provide a highly reliable solution to difficult specialist access, further hindered by the impact of the ongoing pandemic. These obstacles impact specific populations more than others, and broader access to eye care can help unveil true racial variations otherwise attributed to merely genetic differences. Improving medical decisions can also lead to lower eye care cost since the cost of specialist access, referrals, and treatment of advanced eye conditions is very high. As a result, apart from the medical benefits, the implementation of AI also has a strong social aspect, as it could provide solutions to health, clinical, racial, and financial equity.

The utilization of AI in ophthalmic imaging requires the cooperation of a variety of healthcare professionals, including but not limited to physicians, patients, researchers, government officials, and pharmaceutical and imaging device companies. With the collective goal of improving patient care and fostering equity as mentioned above, and with the potential of AI to substantially transform the management of patients, the Collaborative Community on Ophthalmic Imaging (CCOI) was founded in 2019. Experienced experts of the CCOI operate under the values of teamwork, transparency, innovation, and efficiency in a patient-centered approach, strive to resolve any potential issues in eye imaging, and establish the best strategies for the practical use of software in ophthalmology in a way that respects the basic principles of bioethics (325–328).

### The Principles of Artificial Intelligence

The foundation of AI is the completion of tasks by computers through mimicking human (natural) intelligence and cognitive functions. Brain neurons receive signals (input), process that information, and generate results (outputs); these neurons are connected and form networks (neural networks – NNs) capable of complex calculations. These calculations are not static, as the brain can learn from previous data and experience. AI replicates that approach using computer networks, a process referred to as machine learning (ML); inputs are provided to computer models that process them under sets of parameters and give outputs (329). The caveat in that process, which is the basis of supervised learning (SL), is that the real outputs are provided and, accordingly, the model learns to adjust these parameters (training) to calculate the outputs as accurately as possible. Over the past decade, the vast increase in data availability, computer hardware improvement (mostly graphics processing units – GPUs), and the theoretical improvements in NNs have led to an exponential growth of AI.

There are multiple approaches to AI network arrangements, and AI models range from very simple to highly complex. The most simple form of SL is linear regression, where multiple inputs are given to a model that then best adjusts the parameters of each input to provide the most accurate result (330). Logistic regression is an algorithm that adds a sigmoid function to linear regression and displays these results in a probabilistic format (values between 0 and 1) (330). Taking this approach a step further, whereas linear regression tests inputs independently of each other, non-linear regression has the ability to examine interactions between inputs and outputs in several layers (known as multilayer perceptrons), and this is the concept of feed forward neural networks (FFNNs) (330). As the algorithm processes many relationships in several layers, the NN can learn these associations, hence the name deep learning (DL) (331). The metrics of performance for these algorithms are the area under the curve (AUC) in the receiver-operator curve (ROC), sensitivity, specificity, and accuracy.

Specifically in ophthalmology and imaging, NNs have been fine-tuned to process imaging data and are called convolutional NNs (CNNs) (331). Convolution is a mathematical operation that applies filters to images (matrix of parameters) to produce image outputs with different channels (features), whose parameters are continuously tested; this process can be repeated in sequences and actually constructs an FFNN. Inputs can be images in either two- or three-dimensional slices, which is preferred as eye structures between slices are considered as a whole. The final outputs mostly fall into two categories: classification (categorical outputs) or segmentation (image outputs). These can provide both accurate staging of conditions and better-quality images through denoising for clinicians to interpret, since up to 46.3% of SD-OCT scans are prone to artifacts or segmentation errors (330, 332, 333).

### Artificial Intelligence and Glaucoma

Glaucoma is a field that has recently attracted a lot of interest in the integration of AI to ophthalmology and imaging due its involvement of multiple eye structures (anterior chamber angle, iris, retina, and ONH), high prevalence, and reliance on multiple methods to establish a diagnosis. Current clinical assessment of glaucoma relies on a combination of various diagnostic tools to assess anatomy, structure, and function, such as gonioscopy, fundus examination of the ONH, tonometry, OCT scans, and perimetry (a visual field) testing, with none being totally sensitive or specific of its own (325, 334). The interpretation of these results also varies among clinicians (335).

The AI can significantly aid in differentiating glaucomatous from healthy eyes. On this matter, emphasis should be given on moderate glaucoma, where symptoms of early vision loss are apparent or very likely (327). DL algorithms can provide segmentation-free image analysis to quantify relevant structures and can even perform better than traditional segmentation approaches of retinal layers (332, 336). For structural assessment, algorithms could detect changes and signs of optic neuropathy from fundus pictures with greater sensitivity and specificity than clinicians (up to 96.2 and 97.7%, respectively); this can be advantageous, given the subjective and inconsistent interpretation by physicians (325, 337–340). AI can be used on fundus pictures to also differentiate glaucoma from other pathologies of the optic disc, such as papilledema, ischemic optic neuropathy, optic nerve atrophy, compressive optic neuropathy, hereditary optic neuropathy, hypoplasia, and toxic optic neuropathy (341–343). The standard modality of assessing structure, though, is OCT imaging; algorithms can provide assessment of the anterior chamber angle as well as segmentation of the RNFL adjusted for other parameters (age, gender, and eye biometry metrics) to improve the accuracy of the measurements (344–346). Studies have focused on many parameters of the retina and ONH (RNFL, prelaminar area, RPE, choroid, peripapillary sclera, Bruch membrane opening, and minimum rim width), and their performance was highly accurate in identifying glaucomatous eyes (>94%); AI analysis of OCT-A vascular abnormalities of the ONH also yields excellent results (347–354). When comparing various ML classifiers, Wu et al. showed that ganglion cell layer measurements were important in early glaucoma detection, whereas RNFL metrics were more useful during disease progression; in fact, Shin et al. showed that wide-field SS-OCT scans can even outperform the conventional parameter-based methods (355–357). In a recent meta-analysis, including data from numerous studies, Wu et al. reported remarkable overall performance in detecting glaucoma from both fundus pictures (AUC, 97%) and OCT (AUC, 96%), with similar outcomes in classifying glaucoma as well (358). Aside from layer segmentation, the analysis of raw unsegmented OCT volumes (feature agnostic approach) of the ONH has been shown to provide better results than classical ML techniques (AUC of 94% versus 89%) (359). The existing issues with AI applications to OCT, though, are the potentially poor image quality, limited generalizability of certain algorithms to multiple devices and patient-specific factors (anatomy and comorbidities) undermining OCT thickness values (325). To detect functional changes in the visual fields, CNN algorithms have been developed by various teams that mark visual field tests as either normal or glaucomatous with high precision (87.4–87.6%) (360, 361).

A very attractive approach is to use AI to combine structural information with functional outcomes. Glaucoma diagnosis is improved when using both ONH parameters and VF outcomes: algorithms capable of predicting 10-2 VF parameters from macular OCT scans, and 24-1 VF parameters from both macular and ONH OCT scans have been developed (362–365). Also, various teams (Lazaridis et al., Christopher et al., Datta et al., and Xiong et al.) have recently developed algorithms (RetiNerveNet and FusionNet) capable of using both OCT metrics (various layer thickness values) and raw OCT or fundus images to predict VF changes with high accuracy (366–370). Overall, combination of structural and functional information has been shown to outperform structural or functional assessment alone.

These results are especially encouraging when it comes to screening for glaucoma; these imaging techniques are simple and inexpensive and would allow for identification of glaucoma at early stages. As opposed to diagnosis at a more advanced stage, early treatment initiation can both prevent irreversible vision loss and avoid expensive, invasive techniques used for later glaucoma stages, as the cost of management increases with glaucoma progression; its performance, however, still needs to be improved (327). The implementation of DL to AS-OCT is a field-attracting attention; Li et al. have very recently developed a novel 3D deep-learning-based digital gonioscopy system that identified angle closure suspects and that could be used as a screening method for primary angle closure glaucoma (371–376).

AI can also be a valuable tool in establishing the prognosis of glaucomatous progression. Functionally, Wen et al. reported that AI can estimate VF loss up to 5.5 years in the future with minimal error, given only a single test as a starting point (377). Algorithms are also capable of identifying slow disease progression consistently earlier than conventional methods, too (3.5 years versus >3.9 years) (378). Structurally, Christopher et al. reported that RNFL analysis can also predict glaucoma progression more accurately than VF testing (95% versus <86%), and with less error than standard linear regression models (325, 379). Sedai et al. have developed multimodal models using a combination of clinical, structural, and functional information to predict RNFL changes in healthy subjects, patients with glaucoma patients, glaucoma suspects with greater performance than standard linear trend-based estimation (380). These predictive algorithms, however, have not yet been implemented clinically.

### Artificial Intelligence and Age-Related Macular Degeneration

With more than 200 million people affected worldwide, AMD constitutes the most common cause of blindness in developed countries (381). Although no AI device has yet been made commercially available for AMD yet, several algorithms to potentially aid physicians’ decisions do exist.

Up to 25% of patients with AMD can remain undiagnosed by primary care providers, and the use of AI would not require an advanced skillset to operate or retinal expertise (328, 382). An ideal method of AMD screening should be able to efficiently detect AMD and distinguish it from other similar diseases, be cost-effective, and autonomous. Hence, the low cost and simplicity of fundus photographs make it a great candidate for wide AMD screening; models that can classify AMD based on the need for treatment have been developed, which function with great accuracy compared to professional graders (up to 92%) (383). This distinction is important when evaluating algorithms, as early treatment can significantly prevent vision loss in select cases of AMD (large drusen and intermediate AMD, CNV), and the cost of error is high for missing wet AMD; high sensitivity is, therefore, preferable (384). Macular OCT scans have the advantage of depicting the pathological findings of AMD in 3 dimensions and with high resolution; hence, they can be utilized as a next step, following the initial screening with fundus photographs to rule out false positive cases and further classify the true positives (385). De Fauw et al. have developed a screening algorithm capable of identifying multiple retinal conditions, which was able to outperform retinal specialists on both screening success and avoidance of severe and costly errors (386).

Models can also substantially assist physicians in establishing diagnosis of AMD. This can sometimes be challenging, as AMD findings can potentially go unnoticed or appear similar to other retinal conditions (polypoidal choroidal vasculopathy, macular dystrophies, CSR, and others) (328, 384). For this purpose, a variety of algorithms has been offered, focusing on different imaging modalities (fundus pictures, OCT and OCT-A, FA) to detect multiple pathological findings (drusen and pseudodrusen, intra- and subretinal fluid, GA); similar to DR, structural and vascular biomarkers are also utilized for AMD (387–392). As a representative example, Yan et al. were able to utilize a model for identification of drusen, inactive and active CNV with high precision (84.3–97.7%) and AUC (94.–99.%) (393). Keenan et al. also used SD-OCT data across 10 years from the AREDS2 study to construct a model capable of identifying retinal fluid with high accuracy, sensitivity, and specificity compared to retinal specialists (85.1%, 82.2%, 86.5% versus 80.5%, 46.8%, 97.%, respectively) (394).

Even though the use of AI tools in the context of AMD has not been fully optimized, tools capable of prognosis and monitoring the condition are starting to emerge. Algorithms can perform better than specialists in some cases, but their accuracy can be improved; classification accuracy has been reported to be up to 76% and 5-year prognosis accuracy up to 86% in recent studies (395, 396). For classification, Peng et al. developed a model (DeepSeeNet) that more accurately classified AMD, when compared to retina specialists (397). This is especially important in the setting of edema in wet AMD, where anti-VEGF injections are indicated; Potapenko et al. have recently trained a CNN identifying retinal edema with accuracy of 90.9% (398). Even more so when combined with HH-OCT, algorithms can be proved to be a powerful tool in self-monitoring of the condition and more easily identify patients in need of antiangiogenic treatment. As to predicting the disease course, algorithms will help tackle issues, such as predicting conversion to wet (neovascular) AMD, personalize anti-VEGF treatment and predict their response, and increase the use of supplements to decrease the rate of progression. Sarici et al. have recently reported a set of outer retinal biomarkers (ellipsoid zone and RPE attenuation and thickness) useful to predict evolution of dry AMD to subfoveal GA, while Abdelfattah et al. used drusen volume to predict development of wet AMD within 2 years (399, 400). Finally, tools that could be utilized in predicting response to anti-VEGF treatment have also been developed with accuracy comparable to retinal experts (65.4% vs. 53.8–76.9%) (401). A detailed review of tools for AMD progression prediction by fundus photographs or OCT has been constructed by Romond et al. (402).

The implementation of AI on AMD care still faces some challenges. The limitations of OCT (high cost, artifacts, low signal strength, and poor focus) still apply in this field. There is also a great need for dataset diversity in terms of age, race, and socioeconomic status in data analysis to provide broader and more accurate AI outputs, as baseline factors and phenotypes can vary among populations (389, 403).

In general, the impact of AI tools in the setting of AMD can be proved useful in screening, diagnosis, and differentiation of similar appearing conditions, prognosis, and disease monitoring. Whether or not they can benefit physicians and potentially influence clinical decisions remains to be seen.

### Artificial Intelligence and Diabetic Retinopathy

Using AI in the setting of DR is one of the most promising applications in medicine. As opposed to general monitoring of diabetes, which can be performed easily (blood glucose testing, HbA1c levels and others), DR requires qualitative evaluation.

The modalities more commonly used for diagnosis and monitoring of DR are fundus pictures, OCT, and OCT-A scans (as mentioned above). AI has the potential to detect disease in early (even asymptomatic) stages, classify it, predict the disease course, and thus guide treatment in select eyes (404–406). Tools can match or even outperform physicians and can make access to screening broader and less expensive; algorithms and devices are already clinically available (IDx-DR by Digital Diagnostics, Coralville, IA, United States; SELENA+ by EyRIS, Singapore) and have been authorized for use in multiple fundus cameras (407–412). Training models for AI has been steadily increasing for diagnosing DR from fundus pictures, with accuracy, sensitivity, and specificity improving over time (reaching 95.7, 97.5, and 98%, respectively) (412–416).

Similar results are also seen when using CNNs for DR severity grading; Ryu et al. have recently developed a fully automated algorithm to classify DR stages with accuracy of 91–98%, sensitivity of 86–97%, and specificity of 94–99% (417, 418). Application of these models can also establish biomarkers useful for diagnosis and treatment response; these could be structural (retinal layer measurement, hyperreflective foci) or vascular (areas of non-perfusion, vascular leakage, microaneurysm count, and neovascularization) (387).

## Teleophthalmology and Smartphone Fundus Imaging

Telemedicine is defined as “the use of electronic information and communications technologies to provide and support health care when distance separates the participants” (419). In response to its growing demand in recent years, further enhanced by the COVID-19 pandemic, ophthalmologists have begun to implement techniques and technologies more widely to better facilitate patient care in a remote setting. This would make access to eye care more accessible, easier and more convenient for patients, faster, and more cost-effective. In terms of ophthalmic care, teleophthalmology is mostly applicable to ophthalmic emergencies, screening, and monitoring of chronic conditions.

One such technique is imaging of the eye with a smartphone (smartphone imaging – SI). The use of smartphones for clinical imaging in ophthalmology was first introduced by Lord et al. in 2010 (420). It was demonstrated that an iPhone could be used to capture external photos of the orbit and surrounding structures, indirectly image the anterior segment and the fundus of the eye when used with a slit lamp fitted with a 78D lens or a handheld 20D lens (420).

Since its introduction, external attachments and phone applications that offer features, such as image storage and improved user-interface, have been developed with the aim of improving the image quality and utility of SI. Although many variations and distributors exist, a commonly used attachment is a macro lens that can be clipped over the camera of a smartphone to provide supplemental co-axial illumination, making imaged features of the eye more distinguishable (421). The ability for smartphones to be paired with additional attachments and other devices has given rise to techniques that make it applicable in a wider range of clinical scenarios than most other forms of imaging (421).

The current capability of SI in the observation of anterior segment features is promising. Refined techniques using either gonio or macro lens combinations make it possible to capture high-quality videos and standstill images of the iridocorneal angle (422, 423). A trial conducted by Pujari et al. found that inferior angle measurements acquired using the iPhone-macro lens combination demonstrate excellent correlation with measurements acquired using AS-OCT (424). SI can also be used for the analysis of globe torsion. Using a 360° protractor application, the axis of the eye can be compared between smartphone-macro lens images before and after surgical intervention to quantify torsion of the iris and retina (425, 426). It is possible to perform pupillometry using SI, giving smartphones additional utility in the management of neuro-ophthalmological disease (427, 428). McAnany et al. observed excellent agreement when comparing smartphone pupillometry with infrared camera pupillometry, and found significant correlations in pupillary light reflex and re-dilation size between both methods (429). Additionally, methods have been developed for smartphone photography to be used in measuring implantable contact lens vault and assessing the intraocular lens alignment of patients (430–432).

With respect to posterior segment features and pathology, SI can be used to assess and monitor the fundus of patients. While studies have outlined successful techniques for imaging of the optic disk with smartphones (Figure 8) (433–435), Pujari et al. document a strategy to evaluate the ONH using a macro lens phone attachment and 90D handheld lens (436). With a combination of a battery, an LED light source, a barrier, and an excitation filter fitted to an iPhone, Suto et al. also demonstrated for the first time that fundus FA can be captured with a smartphone, producing images comparable to those obtained by indirect ophthalmoscopy (437). More recently, Sivaraman et al. have come forward with a smartphone-coupled device capable of capturing widefield images of the retina beyond the posterior pole, with a field of view of 65° with a single take (438).

The SI is suited for the screening of common diseases, such as glaucoma, AMD, and DR (439–441). SI-based screening for glaucoma, using frequency doubling technology and a head-mounted display, was found to be comparable to Humphrey VF testing with good agreement and correlation between both techniques (440). Photographs of the ONH can also be acquired with SI, with moderate agreement with in-person evaluation and respectable positive and negative predictive values (77.5 and 82.2%, respectively) (441–443). Home monitoring of IOP with rebound tonometers or contact lenses is also possible (441, 444, 445). Teleophthalmology in the setting of AMD screening mostly aims to detect conversion from dry to neovascular form; Li et al. were the first group to demonstrate similar wait times between remote screening in tertiary clinics and referral to retinal specialists, although with increased wait times for treatment initiation (446). Monitoring for AMD progression with Amsler grids or macular VF testing (for example, a ForeseeHome device by Notal Vision Ltd., Tel Aviv, Israel) is more promising and can be beneficial for high-risk patients (441, 447). For DR, screening can be accomplished with imaging modalities, such as non-mydriatic ultrawide-field and multifield fundus photographs, and has been proved to be reliable and cost-effective; Tan et al. compared smartphone ophthalmoscopy to standardized techniques and found that SI had an overall sensitivity of 87% and specificity of 94% (441, 448–450).

Like with many other current ophthalmic imaging modalities, the future of SI may lie in AI. Algorithms, such as AlexNet, EyeArt, and Medios, are accessible and can be coupled with SI for the screening for disease (451). Studies have demonstrated that SI analysis performed using AI is able to improve the sensitivity and specificity of SI in diagnosing retinal disease (452, 453).

## Fluorescence Lifetime Imaging Ophthalmoscopy

The retina exhibits intrinsic autofluorescence: reactive to light stimulation, chemical compounds absorb photons, and promote electrons to a higher power state that subsequently return to their stable state, emitting red-shifted protons in the process. The intensity and patterns of fluorescence can be detected with high-sensitivity detectors in fundus cameras and ophthalmoscopes, mapped and used to diagnose and monitor many macular conditions (454). Since the majority of the signals originate from lipofuscin in the RPE, its high intensity predominates and shadows other retinal molecules that also emit fluorescence. The new technique of FLIO measures the decay lifetime of retinal fluorophores (FLIO lifetimes – FLTs), which are unique to molecules (Dysli et al. have described the lifetimes of each fluorophore in detail) and, therefore, more sensitive of weak fluorophores that are masked using fundus autofluorescence (FAF) (455–457). Hence, FLIO can reveal not only structural but also metabolic and biochemical changes in the retina.

**FIGURE 7:**
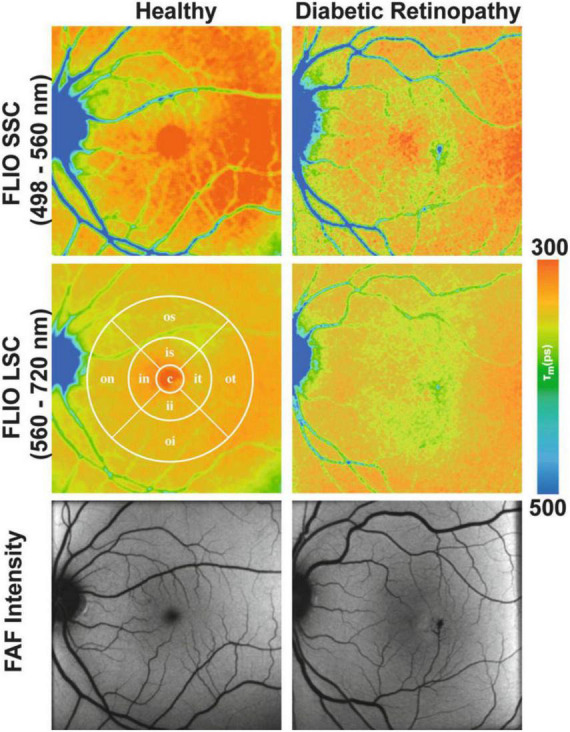
Funds autofluorescence (FAF) lifetime images (FLIO) and FAF intensity images in diabetic retinopathy (DR). Mean funds autofluorescence (FAF) lifetime images (FLIO) from two spectral channels, as well as FAF intensity images from the retina of a healthy control **(Left)** and a diabetic retinopathy patient **(Right)**. The middle left panel comprises a standardized ETDRS grid. This figure was reprinted from Bernstein et al. (455) with permission.

In a clinical setting, the first FLIO device used was developed by Heidelberg Engineering in 2012 and has been shown to be highly reproducible (458–460). FLIO patterns have been identified for a variety of retinal diseases and can be valuable in early detection and detailed monitoring. In AMD, FLIO displays a characteristic pattern of ring-shaped prolongation of the FLTs around the fovea, which is present in the early stages of the disease even before the appearance of drusen and increases as the disease progresses (greater in advanced AMD) (461). Apart from this sign, areas of GA and drusen also display FLT prolongation, and this information could be proved useful in the understanding of their pathogenesis and metabolism as well as monitoring their development (461–463). Since AMD can appear similar to Stargardt disease, FLIO can be used to differentiate the two, since FLTs in Stargardt disease are not prolonged, and the typical ring pattern of AMD is not present. Most importantly, retinal flecks in Stargardt disease appear in FLIO about a year earlier than in other imaging methods like FAF (464). In the case of hydroxychloroquine toxicity, where early toxicity detection is challenging, FLIO could provide a better alternative to electroretinogram (ERG) or OCT, since it can detect prolonged FLTs before structural changes appear (465, 466). FLIO has also been used to identify changes in other retinal conditions as well, including DR (Figure 7), vascular occlusive diseases, CSR, choroideremia, RP, and macular holes, and is believed to be a promising diagnostic method (463, 467–473).

**FIGURE 8:**
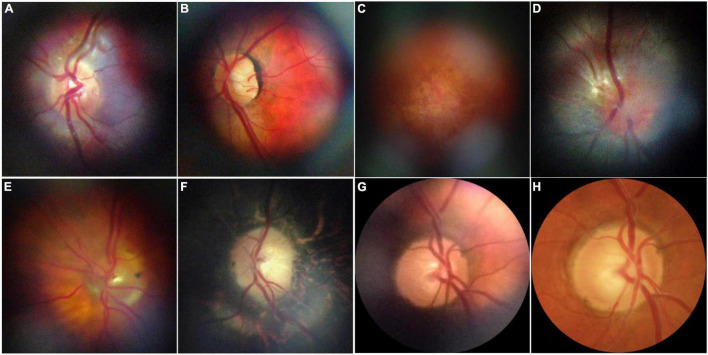
Representative retinal images taken with D-eye. **(A)** A normal optic disk in an undilated child. **(B)** A normal posterior pole in a dilated 29-year-old woman. **(C)** Dry age-related maculopathy in an undilated 75-year-old man. **(D)** Optic nerve glioma in a 23-year-old undilated woman. **(E)** Posterior vitreous detachment in a dilated 72-year-old pseudophakic woman. **(F)** Waxy disk pallor and pigmentary changes in a 50-year-old man with retinitis pigmentosa **(G,H)**. Depiction of the same optic nerve head by D-Eye and Canon CR-2 Retinal Camera. This figure was reprinted from Russo et al. (434) with permission.

## Multimodal Imaging

Multimodal imaging involves the incorporation of two or more imaging technologies for a single purpose. Combinations of imaging modalities make it possible to perform a more comprehensive examination of tissue by compensating for the individual limitations of a single device. Multimodal imaging is often used to improve the utility of commonly used OCT technologies.

The OCT-A is an example of a modality that greatly benefits from multimodal imaging. Although it provides significant clinical utility through its ability to capture the vasculature of the retina, limitations in the acquisition speed of OCT-A create prolonged susceptibility to motion artifacts, and other reductions in image quality. Combining OCT-A, as well as other OCT variations, with SLO (OCT-SLO) allows for the implementation of motion tracking to compensate for involuntary eye movements during imaging (474–477). Commercially available systems that already currently use integrated OCT-SLO technology include the Zeiss PLEX Elite, Heidelberg Spectralis, and Optos Silverstone. Handheld OCT-SLO devices have also been implemented and have expanded the accessibility of multimodal imaging for pediatric, bedridden, and immobilized patients (143, 144). Additionally, OCT-A devices can be combined with vis-OCT to establish a complementary endogenous contrast, which allows for blood oxygen saturation to be quantified and used as a biomarker for DR and AMD (96, 102, 105, 106, 478–482).

While no clinical system is commercially available, photoacoustic microscopy (PAM) is an imaging modality that can be used in ophthalmology to detect the distribution of emitted acoustic waves in vascular tissue, with the ability to map blood absorption without the use of exogenous contrast. Combining PAM with OCT imaging establishes complementary structural and vascular contrast, which has been used to capture neovascularization associated with DR and wet AMD in animal models (483–487). Nguyen et al. further demonstrated that the multimodal combination of PAM with OCT has utility in monitoring vascular and structural changes associated with vascular occlusion (488, 489). PAM has additionally been combined with Doppler OCT to measure retinal oxygen metabolism with the potential to aid in an earlier diagnosis of DR, AMD, and glaucoma (478, 490).

Other experimental imaging techniques that have begun to make headway in multimodal research include polarization-sensitive OCT, photothermal OCT, and optical coherence elastography, which, when combined with more standard techniques, have been shown to allow for the differentiation between depolarizing and birefringent tissue (491, 492), establishment of molecular contrast (493–496), and biomechanical assessment of tissue, respectively (497, 498).

## Conclusion

**FIGURE 9:**
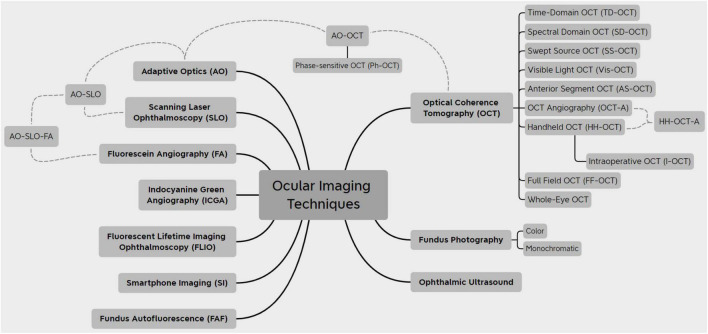
A summary of the modern ocular imaging modalities.

The field of ocular imaging is rapidly advancing. The sheer number of imaging modalities that exist nowadays provides physicians and researchers with a substantially high number to study eye conditions and gather information. This variety of available technologies (Figure 9) provides a multimodal approach to eye imaging, which will inevitably lead to optimization of imaging techniques for each condition individually.

Most innovations are centered around OCT, since it has become the gold standard of managing most retinal diseases; a summary of the OCT imaging modalities is displayed in Table 1. As hardware components and image processing improve, OCT is bound to be improved in multiple ways: faster and higher quality scanning, lower costs, and greater population coverage. These are applications that can be applied worldwide and elevate the standard, commercially available OCT scanner. Some of the new technologies described previously, such as AS-OCT, I-OCT, and HH-OCT, are beginning to be used more widely and are already making a significant impact on medical decisions. Others, like Vis-OCT, FF-OCT, and AO-OCT, that are still rapidly evolving, will undoubtedly be improved and optimized for routine clinical use.

**TABLE 1:** Review and evolution of optical coherence tomography (OCT) imaging technologies in chronological order (3, 195, 196, 252, 255, 265, 308, 312, 499–506).

OCT technology	Year introduced	Commercial availability	Axial resolution in tissue (μm)	Lateral resolution in tissue (μm)	Maximum scanning rates (A-scans per second)	Major clinical application(s)	Advantages	Disadvantages
Time-domain OCT (TD-OCT)	1991	Yes	1.7–15	15–20	400	Most retinal pathologies.	Non-contact, non-invasive.	Low image acquisition speed. Poor spatial resolution.
Anterior segment OCT (AS-OCT)	1994	Yes	1.0	15	2,000,000	Anterior segment conditions (dry eye disease, corneal pathologies).	Detailed imaging of most structures of the anterior segment (corneal layers and precorneal tear film, outflow system, anterior chamber).	
Spectral domain OCT (SD-OCT)	2001	Yes	5–8	6–20	100,000 (clinical)	Most retinal pathologies.	Higher imaging speed and sensitivity than TD-OCT. Capture of 3D volumetric data *in vivo.* Retinal layer segmentation.	Imaging artifacts (projection, motion). Segmentation errors.
Full-field OCT	2002	No	5.6	1.7–2.4	40,000,000 (research)	Ocular surface conditions (dry eye disease, corneal inflammation).	Stable phase, no motion artifacts. Higher scanning power supported. Less sensitive to optical aberrations and signal loss.	Eye motion makes scanning difficult. Difficulties with optical path matching. Post-processing and image averaging necessary. Only select retinal layers visible.
Visible light OCT (Vis-OCT)	2002	No	1–1.4	4.6–10	30,000 (research)		Vastly improved axial resolution. Smaller bandwidth for same resolution. Higher image contrast. Oximetry and calculation of circulation metabolic parameters.	Slow imaging.
Adaptive optics OCT (AO-OCT)	2004	Yes	5–8	2–3	1,000,000 (research)		Vastly improved lateral resolution. Improvement of speckle width. Increased SNR. Visualization of single retinal cells and their function (phase-sensitive OCT). Improved lamina cribrosa visualization.	Slow imaging. Multiple scans required for registration. High cost. High complexity of devices. Limited scanning area. Large data size.
Handheld OCT (HH-OCT)	2007	Yes Yes	3–6	8–15	32,000 (clinical). 350,000 (research).	Pediatric conditions (congenital and pediatric glaucoma, macular edema, macular hole, epiretinal membrane, retinoschisis, retinal dystrophies). Intraoperative OCT (see below).	Imaging of challenging patient populations (bedridden and postoperative patients, children, remote access). Less expensive than benchtop OCT. Imaging of anterior and posterior segments in quick succession.	Probes still connected to bulky mobile carts.
Intraoperative OCT (I-OCT)						Glaucoma surgeries (trabeculectomies, drainage surgeries, canaloplasty, sclerectomy, and angle surgeries). Cornea surgeries (DALK, DSEK/DSAEK, DMEK). Cataract extraction surgeries. Retinal surgeries (ERM peeling, macular hole repair, gene therapies).	Live imaging feedback during surgery. Valuable information on diagnosis and surgery planning. Confirmation of optimal outcome. Assessment of intraoperative complications.	Technician often required. Pausing of surgery sometimes necessary.
Swept source OCT (SS-OCT)	2012	Yes	8–9	20	200,000 (clinical). 6,700,000 (research).	Most retinal pathologies.	Increased SNR. Improved scan quality. Improved imaging of deeper structures.	
Whole-eye OCT	2012	Yes	12.4–19	73	50,000–580,000 (clinical).	Biometry. Surgery planning (cataract extraction and refractive surgeries). Identification of high myopia causality.	Assessment of the entire ocular anatomy with a single scan in standard fields of view.	Time gap for switching scan configurations between anterior-posterior segment
OCT angiography (OCT-A)	2015	Yes	5	15–24 8–16 (ultra high-definition)	200,000 (clinical).	Conditions involving vasculature damage or neovascularization (glaucoma, AMD, DR, BRVO).	Lack of extrinsic dye. Vascular network imaging at different depths. Vascular biomarker identification.	No detection of vessel leakage. Imaging artifacts (projection and motion). Visibility of vessels dependent on flow. Low image contrast. Limited area of visualization.

The most exciting prospect of eye imaging is the incorporation of AI. AI is becoming more accessible and broadly studied, and ophthalmology provides the perfect foundation for its rapid evolution. As such, it is fairly safe to assume that ophthalmology will be among the first medical specialties to transition from a traditional, physician-only care approach to a collaboration between human and software decision-making. The ability to provide valuable data from simple images can help millions of people get eye care in the first place but also improve and optimize the way patients are managed and treated. There are still, though, issues to be considered before safely applying AI in the routine care; these range from mostly technical, namely, the issue of performing AI tasks in most imaging devices, to medical in order to ensure its efficacy and reliability, as well as ethical. Despite these hurdles, AI will be a huge step toward ultimately decreasing blindness and providing equal health care across the entire population.

In conclusion, the evolution of ocular imaging is truly fascinating. The next years will be critical in its evolution and will definitely contribute to the ultimate goals of minimizing blindness and ensuring optimal care for patients.

## Author Contributions

JS and PA contributed to conception and design of the study. JS, PA, and CM acquired the data and performed the analysis. PA and CM wrote the first draft of the manuscript. JS, PA, CM, and GW contributed to manuscript revision and approved the submitted version and took responsibility for the integrity of the data and the accuracy of the data analysis. All authors contributed to the article and approved the submitted version.

## Conflict of Interest

JS: Aerie Pharmaceuticals, Inc. and Opticient: consultant/advisor and equity owner. BrightFocus Foundation and National Eye Institute: grant support. Boehringer Ingelheim, Perfuse, Inc., Regeneron, Inc., and SLACK Incorporated: consultant/advisor. Carl Zeiss Meditec: patents/royalty/consultant/advisor. Massachusetts Eye and Ear Infirmary and Massachusetts Institute of Technology, New York University, Tufts University, and University of Pittsburgh: intellectual property. Ocugenix: equity owner, and patents/royalty. Ocular Therapeutix, Inc.: consultant/advisor and equity owner. The remaining authors declare that the research was conducted in the absence of any commercial or financial relationships that could be construed as a potential conflict of interest.

## Publisher’s Note

All claims expressed in this article are solely those of the authors and do not necessarily represent those of their affiliated organizations, or those of the publisher, the editors and the reviewers. Any product that may be evaluated in this article, or claim that may be made by its manufacturer, is not guaranteed or endorsed by the publisher.

## Abbreviations

AI: artificial intelligence
AMD: age-related macular degeneration
AO: adaptive optics
AUC: area under the receiver-operator characteristic curve
CNV: choroidal neovascularization
CSR: central serous retinopathy
DALK: deep anterior lamellar keratoplasty
DED: dry eye disease
DL: deep learning
DR: diabetic retinopathy
FA: fluorescein angiography
FAF: fundus autofluorescence
FD-OCT: fourier-domain optical coherence tomography
FFNN: feed forward neural network
ILM: internal limiting membrane
IPL: inner plexiform layer
GA: geographic atrophy
GCL: ganglion cell layer
INL: inner nuclear layer
IOP: intraocular pressure
IPL: inner plexiform layer
LDH: laser doppler holography
ML: machine learning
NIR-OCT: near-infrared optical coherence tomography
NN: neural network
ONH: optic nerve head
OCT: optical coherence tomography
OCT-A: optical coherence tomography angiography
ORG: optoretinogram
POAG: primary open angle glaucoma
RGC: retinal ganglion cell(s)
RNFL: retinal nerve fiber layer
ROP: retinopathy of prematurity
RP: retinitis pigmentosa
RPE: retinal pigment epithelium
SD-OCT: spectral-domain optical coherence tomography
SNR: signal-to-noise ratio
SS-OCT: swept-source optical coherence tomography
TD-OCT: time-domain optical coherence tomography
VF: visual field(s)
Vis-OCT: visible light optical coherence tomography.
